# Cell Death Markers in Children with Immune Thrombocytopenic Purpura: A Preliminary Study

**DOI:** 10.1007/s12288-023-01639-0

**Published:** 2023-02-25

**Authors:** Sohier Yahia, Waleed Eldars, Heba Eldegla, Ahmed K. Mansour, Mouna Guaida, Mohamed S. A. Abdelkader, Yahya Wahba

**Affiliations:** 1 Pediatric Department, Mansoura University Faculty of Medicine, Mansoura, Egypt; 2 Department of Medical Microbiology and Immunology, Mansoura University Faculty of Medicine, Mansoura, Egypt; 3 Clinical Biochemistry, Mansoura University Children Hospital, Mansoura University Faculty of Medicine, Mansoura, Egypt; 4 Pediatric Department, Misr University for Science and Technology Faculty of Medicine, Cairo, Egypt

**Keywords:** BCL2, Caspase 3, Caspase 8, ITP, IVIG, Methylprednisolone

## Abstract

Immune thrombocytopenic purpura (ITP) is an autoimmune disease with possible dysregulation of the apoptotic pathways. We aimed to evaluate the possible role of some apoptotic markers (caspase 3, caspase 8 and BCL2) in the pathogenesis and course of ITP. We investigated some apoptotic markers (caspase 3, caspase 8 and BCL2) using the flow cytometry in 60 children with newly diagnosed ITP, 20 children with chemotherapy-related thrombocytopenia (CRT) and 20 healthy children. We also assessed the effects of intravenous immunoglobulin (IVIG) and methyl prednisolone therapies on the platelet apoptosis in children with newly diagnosed ITP. We demonstrated significantly higher values of caspase 3 in the newly diagnosed ITP group than control and CRT groups, and non-significantly higher values of caspase 8 in the ITP group than the healthy group. After IVIG treatment, the platelet count increased in all patients, and there was a significant decrease in caspase 3 and caspase 8 levels while BCL2 level increased. Regarding methylprednisolone treatment, there was a significant decrease in BCL2 and caspase 8 levels while caspase 3 levels did not significantly decrease. There is a possible role of the caspase dependent cell death pathway of the platelets in the occurrence of newly diagnosed ITP. There is heterogeneity in the apoptotic changes of newly diagnosed ITP children who received IVIG versus those who received methylprednisolone.

## Introduction

Immune thrombocytopenia (ITP) is an autoimmune disease with an incidence ranging from 3 to 5 per 100,000 individuals. In the pediatric age group, ITP is mostly acute self-limiting and induced by viral infections, while in adults the disorder is usually chronic. The disease could be asymptomatic or manifested by easy bruising, bleeding tendency, or blood extravasation from capillaries into the mucous membranes and skin [1, 2].

ITP pathogenesis involves both increased peripheral platelet destruction and suppression of the platelet production [1]. Increased platelets destruction is attributed to immunoglobulin G autoantibodies against platelet membrane glycoproteins. Abnormal T-cells activity represents the stimulus for the autoantibody production [3]. Coating of platelets with IgG renders them more prone to opsonization and phagocytosis by the reticuloendothelial system [4].

Apoptosis is considered the main pathophysiological process that controls the cell life span and removal of the infected or damaged nucleated cells. Although platelets are anucleate cells, they undergo events similar to apoptosis [5, 6]. There are two main apoptotic pathways that could regulate the programmed platelet death: the mitochondrial or intrinsic pathway and the death receptor or extrinsic pathway [7]. The latter is initiated by the ligation of death receptors, belonging to the tumor necrosis factor receptor superfamily, that activate the initiator caspase (procaspase 8) with subsequent activation of the effector caspases by caspase 8, such as caspase 3, 6 and 7 [8]. While the intrinsic apoptosis pathway is regulated by members of the B-cell lymphoma protein 2 family (BCL2), which contain both pro-survival and pro-death proteins that could induce changes in the mitochondrial outer membrane with subsequent efflux of the cytochrome c and other apoptogenic proteins into the cytoplasm. Once released, cytochrome c assembles with the initiator caspase (procaspase 9) and apoptotic protease-activating factor 1 to form the apoptosome, which activates caspase 9 and downstream effector caspases [8].

Murine ITP studies have shown that antiplatelet antibodies could induce apoptotic-like processes, and that the intravenous immunoglobulin (IVIG) treatment could decrease these events [9, 10]. Concerning human ITP, platelet apoptosis was suggested in children with newly diagnosed ITP in the form of activated caspase 3, 8 and 9, which were suppressed by the IVIG infusion [11]. Moreover, another study revealed that platelets from adult patients with chronic ITP expressed higher phosphatidylserine exposure associated with dysfunction of the dendritic cells, although other platelet apoptosis markers could not be demonstrated in this study [12]. A more recent study concerning adults with ITP also confirmed the relevance of platelet apoptosis in ITP [13].

The initial lines of ITP treatment include steroids to suppress the immune system and limit platelets destruction. The dose and mode of administration are determined by the platelet count and presence of active bleeding [14]. IVIG is another drug of choice as it leads to increased platelet counts, besides blockage of the reticuloendothelial system via Fc-receptors and it interferes with the apoptotic processes in platelets [14–17].

To our knowledge, few studies were conducted about apoptosis markers of newly diagnosed ITP in the pediatric age group in humans [11, 18]. Most of the previous studies, concerning apoptosis in ITP, were conducted in animal models or human adults [9, 10, 12, 13].

We conducted our preliminary study to investigate the possible role of the apoptotic markers (caspase 3, caspase 8 and BCL2) in the pathogenesis and course of newly diagnosed ITP, being markers for both apoptosis pathways (caspase 3 and caspase 8 for the extrinsic pathway while BCL2 for the intrinsic pathway). We also evaluated the effects of the treatment on the apoptotic markers of the newly diagnosed ITP children.

## Materials and Methods

### Study Design and Participants

An observational prospective preliminary study was carried out in a university-affiliated hospital from June 2019 to May 2021. We enrolled 60 patients with newly diagnosed primary ITP. We also recruited 20 healthy control children from the general outpatient clinics of same hospital while attending for minor complaints or for following up growth and development. In addition, to control effects due to thrombocytopenia itself, 20 children, with chemotherapy-related thrombocytopenia (CRT), were recruited [11]. The platelet count used to define ITP was below 100 × 10^9^/L [19]. We excluded chronic ITP cases, adult ITP cases, secondary ITP and inherited thrombocytopenia. Patients with comorbidities including diabetes mellitus, uncontrolled hypertension, kidney and liver dysfunction, hepatitis C and B viral infections, human immunodeficiency virus and those who had treatment with corticosteroids during the previous six months were also excluded. Patients who developed serious side effects such as anaphylaxis or uncontrolled hypertension during therapy were also ruled out from the study.

### Data Collection

Data were retrieved from patients’ files including the age, gender, prodrome (e.g. history of preceding fever), clinical signs (such as active bleeding, lymphadenopathy and palpable spleen / liver), preceding drug intake, laboratory investigations (complete blood count, antinuclear antibodies, anti-ds DNA, erythrocyte sedimentation rate, complement 3, and bone marrow aspiration if done) and treatment lines. We also retrieved data about infection markers of human immunodeficiency virus, hepatitis C & B viruses, tuberculosis and helicobacter pylori.

### Assessment of Serum Caspase 3, Caspase 8 and BCL2 by the Flow Cytometry

Patients with newly diagnosed ITP were sampled for flow cytometric analyses at the time of diagnosis prior to treatment and 12–24 h after the last treatment dose (prior to discharge from hospital) [11, 18]. Samples from both control and CRT groups were withdrawn once at the time of enrollment. The apoptosis markers were assessed using the FACS Aria III flow cytometer (BD Biosciences, San Jose, USA). The whole blood lysis method was used, and appropriate combinations of monoclonal antibodies (Becton Dickinson Quanti BRITE products) were used. The used monoclonal antibodies were fluorochrome inhibitors of caspases FITC (Rabbit Anti-Active Caspase-3) and Rbm Ab to caspase 8 (E6) and BCL2 oncoprotein /FITC clone 124 [20–24].

### Randomization of the Newly Diagnosed ITP Group According to the Type of Treatment

Sixty consecutive patients with newly diagnosed ITP were randomly assigned into two subgroups: 32 children (18 boys and 14 girls) received IVIG and 28 children (14 boys and 14 girls) received methyl prednisolone using a simple randomization technique with opaque sealed envelopes. Researchers who assessed outcomes were blinded to the given treatment.

### Statistical Analysis

Data analysis was carried out by SPSS program, version 26. Qualitative variables were expressed as numbers (percent). Kolmogorov-Simonov test was used to test the data normality. Normally distributed data were presented as mean ± standard deviation (SD), while non-normally distributed data were presented as median and range (minimum–maximum). Student's t-tests were used for group comparison. *P* values equal to or less than 0.05 were considered significant.

## Results

### Demographic Data

One hundred children were included and divided into: (a) 60 children with newly diagnosed ITP [28 boys (46.7%) and 32 girls (53.3%)]; their ages ranged between 1–13 years (median = 4 years). (b) 20 children with CRT [6 boys (30%) and 14 girls (70%)]; their ages ranged between 4–9 years (median = 5 years). (**c**) 20 healthy control children [4 boys (20%) and 16 girls (80%)]; their age ranged between 4–10 years (median = 6 years) (Table 1). No statistically significant differences existed between the study groups regarding the age (*P* = 0.29) and the gender (*P* = 0.27). No mortality or serious side effects were reported throughout the study period in patient groups.

**Table 1:** Baseline parameters of the study groups

Parameters	Newly diagnosed ITP group (n = 60)	CRT group (n = 20)	Control group (n = 20)
Age range (years)	1–13	4–9	4–10
Median age (years)	4	5	6
Male:Female ratio	28:32	6:14	4:16
Bleeding (n, %)	22 (36.7)	12 (60)	0
Lymphadenopathy (n, %)	10 (16.7)	13 (65)	2 (10)
Palpable spleen / liver	24/0	12/2	0
Prodrome (n, %)	21 (35)	0	0
Mean platelet count (× 10^9^/L)	43.12 $\pm$ 7.78	45.32 $\pm$ 6.76	180.43 $\pm$ 20.56
Mean hemoglobin (g/dL)	11.5 $\pm$ 1.1	10.3 $\pm 1.2$	12.5 $\pm 1.1$
Mean white cell count (× 10^9^/L)	9.5 $\pm 1.1$	8.9 $\pm$ 0.8	9.6 $\pm$ 0.9

*CRT* chemotherapy-related thrombocytopenia, *ITP* immune thrombocytopenia

### Initial Values of the Apoptotic Markers of the Study Groups

**Fig. 1:**
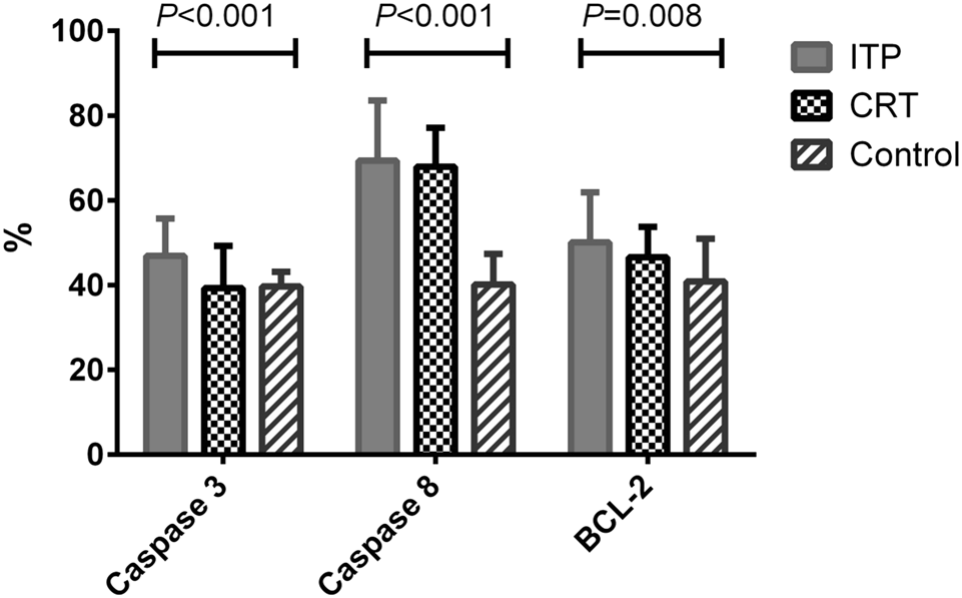
Comparisons between apoptotic markers of the study groups

In the newly diagnosed ITP group, there were significantly higher values of caspase 3 when compared with control and CRT groups (*P* = 0.001 for each). As regards caspase 8, there was insignificant difference between the ITP group and the CRT group (*P* = 0.69) while there were significant differences between the ITP group and the control group (*P* < 0.001) and between the control and CRT groups (*P* < 0.001). As regards BCL2, there was a significant higher value in the newly diagnosed ITP group than the control group (*P* = 0.008), while there were insignificant differences between the CRT group and ITP and control groups (Table 2 and Fig. 1).

**Table 2:** Comparison between the levels of the apoptotic markers and the platelet count in the study groups

Laboratory markers	Newly diagnosed ITP group (n = 60)	CRT group (n = 20)	Control group (n = 20)	*P* value
Caspase 3 (%)	46.89 $\pm$ 8.79	39.31 $\pm$ 9.97	39.7 $\pm 3.44$	P_1_ = 0.001, P_2_ = 0.001 P_3_ = 0.88
Caspase 8 (%)	69.36 $\pm$ 14.19	67.92 $\pm$ 9.18	40.13 $\pm$ 7.21	P_1_ = 0.69, P_2_ < 0.001 P_3_ < 0.001
BCL2 (%)	50.11 $\pm$ 11.74	46.58 $\pm 7.19$	40.88 $\pm 10.08$	P_1_ = 0.25, P_2_ = 0.008 P_3_ = 0.13
Platelet count (× 10^9^/L)	43.12 $\pm$ 7.78	45.32 $\pm$ 6.76	180.43 $\pm$ 20.56	P_1_ = 0.262, P_2_ < 0.001 P_3_ < 0.001

Data were presented as mean ± SD and compared using Student's t-test

*BCL2* B-cell lymphoma protein 2, *CRT* chemotherapy-related thrombocytopenia, *ITP* immune thrombocytopenia, P_1_ represents the newly diagnosed ITP group vs. the CRT group; P_2_ represents the newly diagnosed ITP group vs. the control group; P_3_ represents the CRT group vs. the control group

### The Overall Effect of the Treatment on the Apoptotic Markers of the Newly Diagnosed ITP Group

The newly diagnosed ITP group showed significantly higher values of caspase 3, caspase 8 and BCL2 in the pretreatment state than the post treatment state (*P* = 0.01, < 0.001 and 0.034, respectively) (Table 3).

**Table 3:** Comparisons between pre- and post-treatment values of the apoptotic markers and the platelet count in the newly diagnosed ITP group (n = 60)

Laboratory markers	Pre-treatment values	Post-treatment values	*P* value
Caspase 3 (%)	46.89 $\pm$ 8.79	43.73 $\pm$ 8.35	0.01
Caspase 8 (%)	69.36 $\pm$ 14.19	60.1 $\pm$ 15.8	**<** 0.001
BCL2 (%)	50.11 $\pm$ 11.74	48.5 $\pm$ 11.06	0.034
Platelet count (× 10^9^/L)	43.12 $\pm$ 7.78	162 $\pm$ 21.43	< 0.001

Data were shown as mean ± SD and compared by Student's t-test

*BCL2* B-cell lymphoma protein 2

### The Individual Effects of IVIG or Methyl Prednisolone on the Apoptotic Markers of the Newly Diagnosed ITP Group

In the pretreatment state, there were no significant differences in caspase 3, caspase 8 and BCL2 levels between both subgroups (IVIG and methyl prednisolone). Whereas, there were significant lower values of caspase 3 and caspase 8 levels after receiving IVIG than methylprednisolone (*P* = 0.017 and 0.003, respectively). Whereas, there was a significant lower level of BCL2 after treatment in the methylprednisolone group compared with IVIG group (*P* = 0.036, Table 4).

**Table 4:** Pre- and post-treatment values of apoptotic markers and the platelet count in the newly diagnosed ITP group

Laboratory markers	Treatment state	IVIG (n = 32)	Methylprednisolone (n = 28)	*P* value
Caspase 3 (%)	Pre-treatment	45.97 $\pm$ 7.28	47.94 $\pm$ 10.3	0.39
	Post-treatment	41.36 $\pm$ 9.78	46.45 $\pm$ 5.29	0.017
Caspase 8 (%)	Pre-treatment	66.1 $\pm$ 18.4	73.1 $\pm$ 5.11	0.06
	Post-treatment	54.53 $\pm$ 18.7	66.4 $\pm$ 7.83	0.003
BCL2 (%)	Pre-treatment	49.26 $\pm$ 13.12	51.09 $\pm$ 10.08	0.55
	Post-treatment	51.29 $\pm$ 12.5	45.32 $\pm$ 8.23	0.036
Platelet count (× 10^9^/L)	Pre-treatment	46.43 $\pm$ 8.81	44.54 $\pm$ 7.65	0.382
	Post-treatment	161.32 $\pm$ 21.3	141.35 $\pm$ 19.21	0.0004

Data were shown as mean ± SD and compared by Student's t-test

*BCL2* B-cell lymphoma protein 2, *IVIG* intravenous immunoglobulin

### Apoptotic Markers Values Pre and Post Treatment in IVIG and Methylprednisolone Groups

**Fig. 2:**
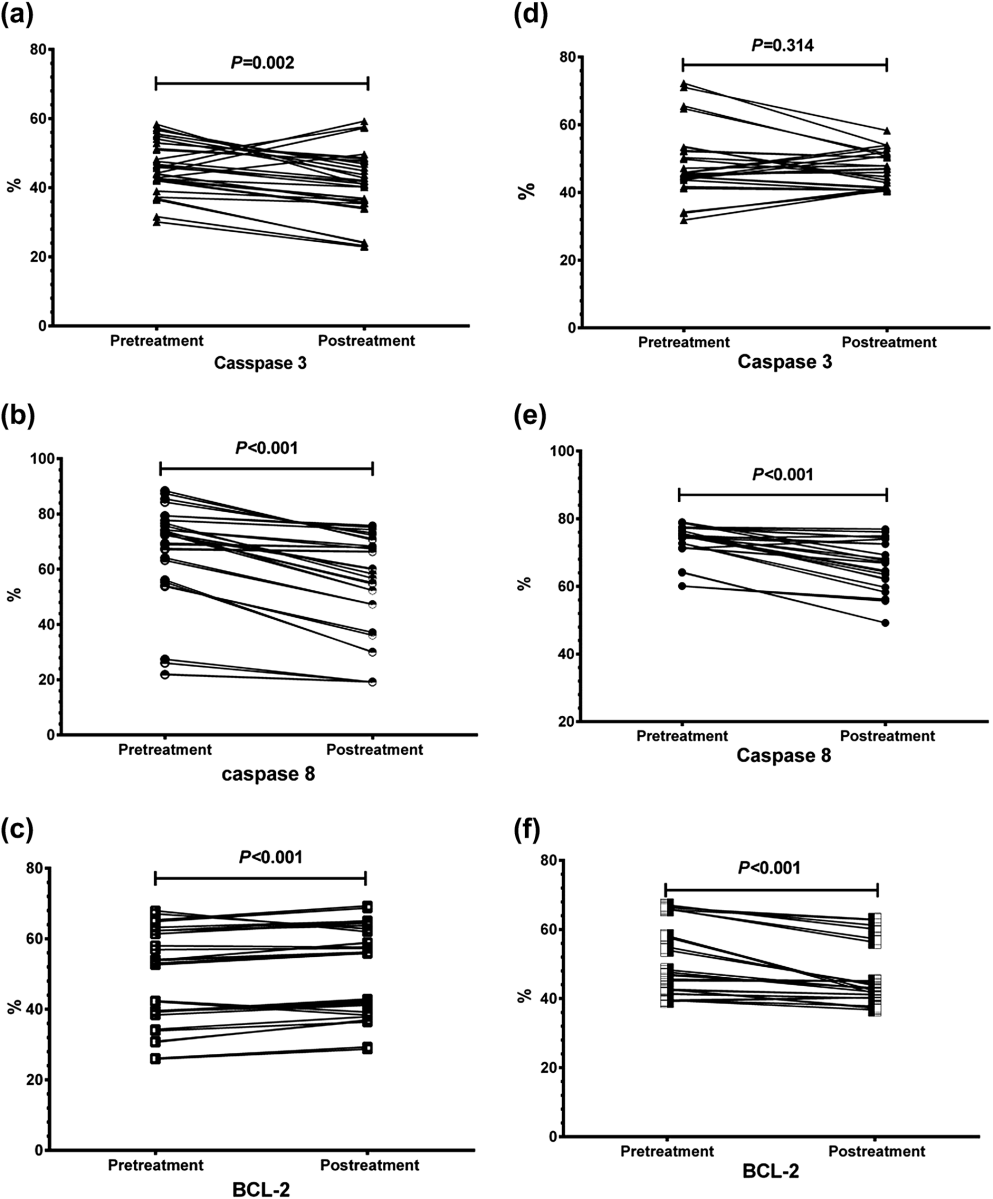
**a–c** Significant reductions in caspase 3 (*P* = 0.002), caspase 8 (*P* < 0.001) and significant increase in BCL2 (*P* < 0.001) in the newly diagnosed ITP patients treated with IVIG. **d**–**f** Significant reductions in caspase 8 and BCL2 (*P* < 0.001), with non-significant change in caspase 3 levels in the newly diagnosed ITP patients treated with methylprednisolone (*P* = 0.314)

Regarding the IVIG treatment, there was a significant reduction in caspase 3 (*P* = 0.002) and caspase 8 (*P* < 0.001) levels after treatment and a significant increase in the BCL2 level after the treatment (*P* < 0.001). While with the methylprednisolone treatment, caspase 8 and BCL2 levels decreased after the treatment (*P* < 0.001). Meanwhile, there were insignificant changes regarding caspase 3 (*P* = 0.314) (Fig. 2).

## Discussion

ITP is an autoimmune disorder with possible involvement of the apoptosis pathways [7].The present study revealed higher levels of all apoptotic markers in the newly diagnosed ITP cases, denoting involvement of both apoptosis pathways. This is in agreement with Speer and Schmugge who reported that platelets undergo apoptotic-like events through translocation and activation of BCL2 family members [25]. In contrary, there is less pronounced caspase activation and resistance to platelet apoptosis in chronic ITP [26, 27].

Newly diagnosed ITP patients, after IVIG treatment, had considerable decrease in caspase 3 & caspase 8 while BCL2 increased. The clinical presentation of ITP receded and the platelet count increased. Meanwhile, after treatment with methylprednisolone, there was significant decrease in caspase 8 and BCL2 while caspase 3 did not significantly decrease.

Regarding BCL2, there was a significant decrease with the methyl prednisolone treatment and a significant increase with the IVIG treatment. These findings indicate heterogeneous changes in the apoptotic ITP pathways, based upon the used treatment. IVIG and methylprednisolone possibly affected platelet apoptosis pathways in different ways. Recognition of these apoptotic changes may open the gate for better understanding of the mechanism of action of these therapies. These apoptosis markers could be possibly used as follow-up markers for the treatment response. But, further extended large scale studies with more apoptosis markers are still needed to confirm our results.

These finding were similar to a previous study that reported a decrease in the proportion of platelets with caspase 8 & caspase 3 after the IVIG treatment [11]. Leytin et al. [9] also confirmed that IVIG prevents downstream apoptotic events and caspase 3 activation. Speer and Schmugge [25] also observed activation of caspase 3 in 40% of the platelets in primary ITP and decrease to 10% after the IVIG therapy. Several studies approved that the apoptotic platelets events such as activation of caspase 3, 8 and 9 increase specifically in ITP but not in CRT and decrease after the IVIG treatment [11, 18, 25].

The strengths of our study included the prospective design. Understanding the effects of two well-known lines of treatment on the apoptotic markers of the newly diagnosed ITP children represents a new add to literature. Previous human reports focused mainly on the effects of the IVIG therapy on apoptosis [11]. However, the current study had some limitations, being a single center study with a relatively small sample size and had limited studied apoptotic markers and a short follow-up period.

## Conclusion

There is a possible role of the caspase dependent cell death pathway of the platelets in the occurrence of newly diagnosed ITP in children. There are heterogeneous changes in the apoptotic markers of newly diagnosed ITP, based upon the used therapy. Levels of caspase decrease when children received IVIG or methylprednisolone treatment but more pronounced with IVIG, while the BCL2 level increases with the IVIG therapy and decrease with methylprednisolone. In the future studies, we recommend extended large scale multicenter studies with more apoptosis markers to generalize our results.

## Data Availability

The datasets used and/or analysed during the current study are available from the corresponding author on reasonable request.
